# Green Infrastructure Offset the Negative Ecological Effects of Urbanization and Storing Water in the Three Gorges Reservoir Area, China

**DOI:** 10.3390/ijerph17218077

**Published:** 2020-11-02

**Authors:** Qipeng Liao, Zhe Wang, Chunbo Huang

**Affiliations:** 1 School of Arts and Communication, China University of Geosciences, Wuhan 430078, China; liaoqp@cug.edu.cn (Q.L.); 18222933115@163.com (Z.W.); 2 Faculty of Fine Art, University of Barcelona, 08028 Barcelona, Spain; 3 Research Center for Spatial Planning and Human-Environment System Simulation, School of Geography and Information Engineering, China University of Geosciences, Wuhan 430078, China

**Keywords:** land use change, landscape planning, ecosystem services, ecological restoration, Carnegie–Ames–Stanford Approach (CASA)

## Abstract

Land use planning usually increases the uncertainties of the ecosystem structures and functions because various human demands usually bring both positive and negative ecological effects. It is critical for estimating various land use changes and their ecological effects, but the previous studies have failed to decouple the respective and the combined effects of different land use changes on ecosystem services. Net primary productivity (NPP) could be used to indicate many ecosystem services such as carbon sequestration and storage. Here, we employed a light use efficiency model to estimate the spatial and temporal dynamics of NPP in the Three Gorges Reservoir (TGR) area from 2000 to 2015, and designed four scenarios to analyze the relative roles of afforestation, urbanization and storing water on NPP dynamics. Our results documented that terrestrial NPP of the TGR area increased from 547.40 gC•m^−2^ to 629.96 gC•m^−2^, and carbon sequestration capacities were 31.66 TgC (1Tg = 10^12^g) and 36.79 TgC in 2000 and 2015, respectively. Climate change and land use change both could contribute to carbon sequestration with 4.08 TgC and 1.05 TgC. Among these land use changes, only afforestation could sequester carbon with 2.04 TgC, while urbanization-induced and impoundment-induced emissions were 0.12 TgC and 0.32 TgC, respectively, and other land use changes also could release 0.55 TgC of carbon. This finding suggested that although positive and negative environmental effects happened simultaneously over the past decades, green infrastructure could effectively offset the carbon emissions from urbanization and storing water in the TGR area, which provides some fundamental supports for further ecological restoration and contributes to empowering land use policies towards carbon sequestration and storage at the regional scale.

## 1. Introduction

Increasing human activities have significantly altered the ecosystem function and service on terrestrial surfaces, especially in ecologically vulnerable areas [1,2]. It is of great significance for regional sustainable development to clarify the ecosystem evolution mechanism against the background of global climate change and intensified human activities [3]. Human activities (e.g., ecological restoration, green infrastructure construction and urbanization) and global climate change have significantly changed the landscape pattern, regional biodiversity and ecosystem functions [4,5]. However, previous studies lack an in-depth analysis of studies related to how land use change (such as green infrastructure construction, deforestation and urbanization) and climate change affect the regional ecological environments. This cannot effectively support the formulation of land use and ecological protection-oriented policies. Decoupling the effects of climate change and land use changes on the ecosystem is an effective means to expose the disturbances caused climate change and human activities on ecosystem services. In this context, it is necessary to analyze the impact of various land use change and climate change on the ecosystem structure and function.

Net primary productivity (NPP) is the total amount of organic matter accumulated by vegetation per unit of area per unit of time and represents the carbon sequestration capacity of vegetation [6,7]. As one of the most critical components of the carbon cycle in terrestrial ecosystems, NPP could be used to indicate many ecosystem services (e.g., carbon sequestration and storage, water conservation) [6,8]. Meanwhile, global NPP could refer to the biophysical limit for the human to live and survive on the earth [9]. NPP is mainly determined by the ecosystem structure, such as the configuration of vegetation [10] and the horizontal and vertical distributions of forests [11]. Many factors such as climate change [12,13], land use transition [14], and human disturbance [15] would change the ecosystem structures, which driving terrestrial NPP dynamics at various spatial scales. Therefore, it is vital to characterize and quantify the spatiotemporal variations of NPP as a result of various driving forces for land use planning and ecosystem regulation.

Numerous studies (e.g., Wu et al. [16], Gingrich et al. [17], Khalifa et al. [18]) have analyzed the spatiotemporal dynamics of terrestrial NPP at the regional scale and documented that human-induced land use change and climate change were among the primary driving forces for NPP dynamics [19]. Although some studies tried to decouple the individual effects of land use change and climate change, there was a large uncertainty for NPP dynamics due to the interaction effects of various land use changes [9,16]. For instance, the implementation of green infrastructure projects and urban sprawling could occur simultaneously at the regional scale [1]. The former such as afforestation or reforestation would increase the terrestrial NPP while the latter could decrease the terrestrial NPP. Therefore, the combined effects of ecological restoration and urbanization are complex and difficult to demonstrate for a regional study [12,20]. Consequently, it is important to quantitatively assess the magnitude and direction of the respective and the combined effects of various land use changes on NPP dynamics.

Generally, NPP could be assessed by field measurements and model estimations [21]. Field measurements are usually used at the local scale and can provide a high-precision NPP evaluation [22,23]. The eddy covariance technique is among the representative measuring methods and could collect valuable data such as carbon flux, water, and energy exchange [24,25]. However, field measurement is spatially limited and likely to be affected by the site conditions of the flux tower site. The NPP estimation models include statistical models [7,26,27], process-based models [28,29,30], and light use efficiency models [31,32,33], which can extrapolate over vast continental areas. The statistical model, with few input parameters and low evaluation accuracy, is generated by analyzing and modeling field measurement [26]. In contrast, the process-based model needs many input parameters and has high evaluation accuracy [34]. The light use efficiency model could spatially explicit information on carbon exchange at various spatial scales and have the greater potential to adequately address the conflict between the input data and the accuracy due to its theoretical basis and practicality [33].

Since the construction of the Three Gorges Dam Project in China, the Three Gorges Reservoir (TGR) area of the Yangtze River has attracted worldwide attention. The world’s largest hydroelectric project could help to generate hydropower, control floods, and improve navigation [35,36], but carbon emissions have worsened due to the hydropower plant and infrastructure construction [37]. Moreover, Chongqing City, a rapidly developing municipality, is located to the southwest of the TGR area. The Western Development Project has promoted urban expansion and accelerated economic growth there, which has also increased carbon emissions in this region [37]. Meanwhile, the TGR area is one of the most ecologically vulnerable areas and suffers many environmental problems such as soil loss [36], nonpoint source pollution [38], and habitat fragmentation [39] because of its rugged topography and intensive human activities. The Chinese government has launched and implemented a series of green infrastructure projects, especially some key afforestation projects, to increase resilience, enhance the provision of ecosystem services, recover biodiversity and reduce the negative ecological effects [1,36], which also contributed to carbon sequestration and storage. Although some studies (such as Chen and Xiao [30], Zhao et al. [40], Xiao et al. [41]) have estimated and analyzed the spatiotemporal dynamics of terrestrial NPP in the TGR area, the overall and relative effects of these land use changes on NPP dynamic was poorly understood.

In this study, we used a light use efficiency model, Carnegie–Ames–Stanford Approach (CASA), to estimate the NPP of the TGR area and explored the spatiotemporal dynamics of NPP from 2000 to 2015. Meanwhile, we designed four scenarios to analyze the relative roles of afforestation, urbanization and storing water on NPP dynamics. Our study could provide fundamental support for further ecological restoration and green infrastructure planning in the TGR area and contribute to empowering land use policies towards carbon sequestration and storage at the regional scale. The specific objectives are as follows (1) estimate and characterize the spatiotemporal variation of NPP; (2) analyze the overall effects of climate and land use change on NPP dynamics; (3) demonstrate the relative effects of various land use changes on NPP dynamics.

## 2. Materials and Methods

### 2.1. Study Area

**Figure 1:**
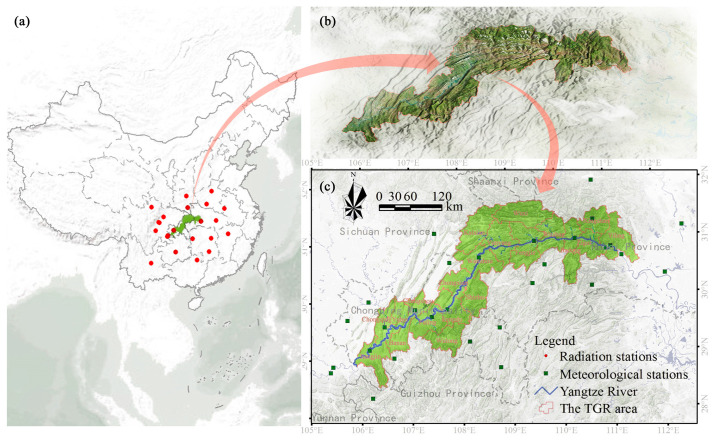
Locations of the radiation stations (**a**), the topographical characters of the Three Gorges Reservoir (TGR) area (**b**) and the meteorological stations of the precipitation and temperature data (**c**).

The TGR area is located in the upper and middle reaches of the Yangtze River (Figure 1), and lies between 28°31′~31°44′ N and 105°50′~111°40′ E. It covers about 5.8 × 10^4^ km^2^ and consists of 20 districts or counties (four in Hubei Province and 16 in Chongqing municipality), which are directly influenced by the construction of the Three Gorges Dam to store water. In this region, the mountainous areas account for more than 74% of the total area, and the hills account for 21.7%, while the small plains alongside river valleys make up only 4.3%. Meanwhile, the steep slope (>25°) accounts for 24.74% of the total area. The TGR area has a humid mid-subtropical monsoon climate, with a mean annual temperature of 20 °C and mean annual precipitation of 1200 mm. It is a typical biodiversity hotspot with rich plant resources in China. However, the historical land use types are mainly croplands because of the long-term anthropogenic activity and dense population.

Over the past decades, the Chinese government has launched a host of green infrastructure projects such as the Grain-to-Green Program, the Natural Forest Protection Program, and the Yangtze River Shelter Forest Project [35]. Forestland has significantly increased, while cropland has sharply declined [1]. These vital projects have actively contributed to promoting forest cover and ecosystem restoration, and the TGR area has gradually changed from an agricultural landscape to a forest landscape.

On the other hand, urbanization has significantly increased the proportion of built-up land due to a series of land use policies and incentives such as the Western Development Project. Meanwhile, storing water has altered the landscape of the TGR area. The Three Gorges Dam began to store water in 2003, and the water level reached 135 m in June 2003, 156 m in October 2006, and 175 m in October 2010 [36]. Impoundment submerged many forestlands and generated a special riparian zone, i.e., an ecozone between aquatic and terrestrial ecosystems, which has significantly influenced ecosystem structure.

### 2.2. Data and Processing

Data used in this study mainly including land use maps, climate data (e.g., precipitation, temperature, and radiation), MODIS-NDVI images, and DEM (Table 1). Land use maps of the TGR area in 2000, 2005, 2010, and 2015 were derived from Landsat TM/ETM+/OLI imageries with 30 m resolution using the supervised classification and artificial neural network methods, provided from our previous study [1,35]. The land use types included forestland (e.g., broad-leaved forest, coniferous forest, shrubby and mixed forest), grassland, cropland, water, built-up land, and bare land.

We collected the monthly radiation data of 22 meteorological stations during 2000–2015 from the National Meteorological Administration of China [1,36]. These meteorological stations distribute in or near the TGR area (Figure 1a). The ordinary kriging method was used to interpolate the monthly total radiation maps with 30 m resolution [36]. Meanwhile, we collected the monthly total precipitation and mean temperature data of 29 meteorological stations (Figure 1c). Considering the tremendous effect of topography on climate, the co-kriging method was used to interpolate the monthly total precipitation and mean temperature maps with 30 m resolution [1]. To obtain the monthly NDVI, the time-series MODIS-NDVI images were processed using the maximum value compositing algorithm at the pixel level [36].

### 2.3. Estimating NPP

#### 2.3.1. CASA Model

As a light use efficiency model, the CASA was widely used to assess the NPP at the regional scale [31]. In this study, we employed it to calculate the monthly NPP (Equation (1)) and estimated the annual NPP of the TGR area by summing the twelve-monthly NPP:

(1) NPP = APAR×ε

where NPP is the net primary productivity (gC•m^−2^), APAR is the absorbed photosynthetic active radiation (MJ•m^−2^•yr^−1^), and ε is the light use efficiency (gC•MJ^−1^).

##### Calculating APAR

Radiation is the vegetation photosynthesis energy source, but not all radiation could be absorbed. We used the interpolated monthly total radiation maps and the monthly NDVI as input data to calculate the APAR by employing the following equations [9,42]:

(2) APAR(x, t)=SOL(x, t)×FPAR(x, t)×0.5

(3) $$\mathrm{FPAR}\left(x,t\right)=\frac{\mathrm{FPAR}{\left(x,t\right)}_{\mathrm{NDVI}}+\mathrm{FPAR}{\left(x,t\right)}_{\mathrm{SR}}}{2}$$

(4) $$\mathrm{FPAR}{\left(x,t\right)}_{\mathrm{NDVI}}=\frac{\left(\mathrm{NDVI}\left(x,t\right)-{\mathrm{NDVI}}_{i,min}\right)\left({\mathrm{FPAR}}_{\max}-{\mathrm{FPAR}}_{\min}\right)}{\left({\mathrm{NDVI}}_{i,\max}-{\mathrm{NDVI}}_{i,\min}\right)}+{\mathrm{FPAR}}_{\min}$$

(5) $$\mathrm{FPAR}{\left(x,t\right)}_{\mathrm{SR}}=\frac{\left(\mathrm{SR}\left(x,t\right)-{\mathrm{SR}}_{i,\min}\right)\left({\mathrm{FPAR}}_{\max}-{\mathrm{FPAR}}_{\min}\right)}{{\mathrm{SR}}_{i,\max}-{\mathrm{SR}}_{i,\min}}+{\mathrm{FPAR}}_{\min}$$

(6) $$\mathrm{SR}\left(x,t\right)=\frac{1+\mathrm{NDVI}\left(x,t\right)}{1-\mathrm{NDVI}\left(x,t\right)}$$

where, APAR(*x, t*) is absorbed photosynthetic active radiation in the geographic of a given location *x* and *t* month. SOL(*x, t*) is the total solar radiation in *x* pixel and *t* month; and FPAR(*x, t*) is the fraction of the photosynthetically active radiation absorbed by vegetation canopy in *x* pixel and *t* month, and could be determined by NDVI. SR(*x, t*) is the simple ratio of NDVI in *x* pixel and *t* month. FPAR(*x, t*)_NDVI_ and FPAR(*x, t*)_SR_ respectively are FPAR calculated by NDVI and SR in *x* pixel and *t* month. FPAR_max_ and FPAR_min_ represent the maximum and minimum values of FPAR in the TGR area, and are 0.950 and 0.001 in this study. NDVI(*x, t*) and SR(*x, t*) are the NDVI and SR in *x* pixel and *t* month, respectively. NDVI*_i,_*_min_ and NDVI*_i,_*_max_ refer to the minimum and maximum values of the NDVI for the land use type of *i* in the *t* month (Table 2). SR*_i,_*_min_ and SR*_i,_*_max_ refer to the minimum and maximum values of the SR for the land use type of *i* in the *t* month (Table 2).

##### Assessing ε

The light use efficiency (ε) is an important parameter for estimating NPP and could be affected by many environmental conditions such as temperature and moisture [31,42]. The interpolated monthly mean temperature maps were used to calculate two temperature stress coefficients, while the interpolated monthly mean temperature and total precipitation maps were used to conduct the moisture stress coefficient. Meanwhile, the maximum light-use efficiency under the ideal condition of different land use types was determined by an empirical method [42,43]:

(7) $$\varepsilon \left(x,t\right)=T_{\varepsilon 1}\left(x,t\right)\times T_{\varepsilon 2}\left(x,t\right)\times W_{\varepsilon }\left(x,t\right)\times \varepsilon_{\max}$$

(8) $$\begin{array}{c} T_{\varepsilon 1}\left(x,t\right)\\ =\{\begin{array}{cc} 0, & \mathrm{if the monthly mean temperture}\leq -10\;{}^{\circ}C\\ 0.8+0.02T_{\mathrm{opt}}(x)-0.0005{\left[T_{\mathrm{opt}}(x)\right]}^{2}, & \mathrm{if the monthly mean temperture}>-10\;{}^{\circ}C \end{array} \end{array}$$

(9) $$T_{\varepsilon 2}\left(x,t\right)=\frac{1.1814}{\left\{1+e^{0.2\times \left[T_{opt}\left(x\right)-10-T\left(x,t\right)\right]}\right\}\times \left\{1+e^{0.3\times \left[-T_{opt}\left(x\right)-10+T\left(x,t\right)\right]}\right\}}$$

(10) $$W_{\varepsilon }\left(x,t\right)=0.5+\frac{0.5\times E\left(x,t\right)}{E_{p}\left(x,t\right)}$$

where, *ε(x, t)* is the light use efficiency in *x* pixel and *t* month. *T_ɛ1_(x, t)* and *T_ɛ2_(x, t)* are temperature stress coefficients, and indicate the reduction of light-use efficiency caused by temperature factor. *W_ɛ_(x, t)* is the moisture stress coefficient, and reflects the reduction of light-use efficiency caused by the moisture factor. *ε_max_* is the maximum light-use efficiency under the ideal condition for different land use types (Table 2). *T_opt_(x)* represents the optimal temperature of the land use type in the geographic of a given location *x*, and could be determined by the mean temperature in the month with the largest NDVI. *T(x, t)* is the monthly mean temperature in *x* pixel and *t* month. *E(x, t)* and *E_p_(x, t)* are the actual evapotranspiration and potential evapotranspiration in *x* pixel and *t* month, which could be determined by temperature and precipitation.

#### 2.3.2. Validation of NPP Estimation of the CASA Model

It is challenging to obtain measured NPP data at the regional scale. Even if these data could be acquired, it seems impossible to ensure that the field data and the remote-sensing images are obtained in the same period. Although it is difficult to verify the NPP estimation accuracy of the model simulation, Wang et al. [32] documented that vegetation productivity is closely related to aboveground biomass. Therefore, the relationship between aboveground biomass and NPP could be conducted to validate the CASA model’s NPP estimation. First, the aboveground biomass in some forest plots was calculated by using the field data according to Zeng et al. [43] in 2000 and 2013. Second, we used the plot boundary as the statistical unit to obtain the NPP estimation of the CASA model for these forest plots in 2000 and 2013. Third, the correlation analysis between the aboveground biomass and the NPP estimation was performed in 2000 and 2013. These significant positive relationships (Figure S1) indicated that the model’s estimation accuracy was satisfactory, and the CASA model could support us to analyze the spatiotemporal variations of NPP in the TGR area.

### 2.4. Scenario Design

To decouple the effects of climate change, afforestation, urbanization, storing water and other land use changes on the NPP dynamic, we designed four scenarios to estimate the potential NPP of 2015 (Table S1). We fixed the precipitation, temperature, and radiation as the level of 2000 in all these scenarios, but changed the NDVI images and the land use map to estimate the potential NPP caused by different land use changes. In scenario A, we removed the effect of climate change and estimated the overall effect of all land use changes on NPP dynamics between 2000 and 2015 (*ΔLUCC*). The effect of climate change could also be estimated because we hypothesize that the NPP was only influenced by climate conditions and land use changes.

Based on scenario A, we designed the other three scenarios: no afforestation (Scenario B), no urbanization (Scenario C), and no storing water (Scenario D). Then, we estimated the individual effects of afforestation, urbanization and storing water on the NPP dynamic and recorded them as Δ*Afforestation*, Δ*Urbanization*, and Δ*Storing water* (Table S1). Meanwhile, Δ*LUCC* consists of their effects and the effect of other land use changes (Δ*Others*), expressed as Equation (11). Therefore, we could also calculate Δ*Others* according to the above four scenarios.

(11) ΔLUCC=ΔAfforestation+ΔUrbanization+ΔStoring water+ΔOthers

### 2.5. Statistical Analysis

#### 2.5.1. Trend Analysis

To display the change trends of the NPP and climate variables (e.g., precipitation, temperature, and radiation) during 2000–2015, we applied the least-square linear regression model to fit these variables for the whole TGR area and at the pixel scale [1,36]. The modeled *slope* was used to describe the change trends because this approach is simple and computationally efficient [1].

#### 2.5.2. Correlation Analysis

As a nonparametric version of the Pearson product-moment correlation, Spearman’s rank correlation analysis was carried out to examine the relationship between the annual NPP and climate variables for the whole TGR area and at the pixel level.

#### 2.5.3. Correspondence Analysis

Correspondence analysis was employed to evaluate the relevance of NPP change with forest change types. This analysis had the potential to identify the relationship between afforestation and NPP dynamics. It was especially useful in light of spatial consistency. In applying correspondence analysis, scores were discretized to place the data into a contingency table [44]. In this study, scores could be discretized into several categories (e.g., increase, decrease, and no change) according to the results of trend analysis for annual NPP and forest coverage in the 1km grid, and then newly categorized data can be analyzed with biplot embedded in the correspondence analysis. The biplot analysis embedded in correspondence analysis can visually examine the relationships between row and column categories in a plane constructed by a pair of dimensions to enhance understanding of the category relationships [44].

## 3. Results

### 3.1. Dynamic of NPP from 2000 to 2015

**Figure 2:**
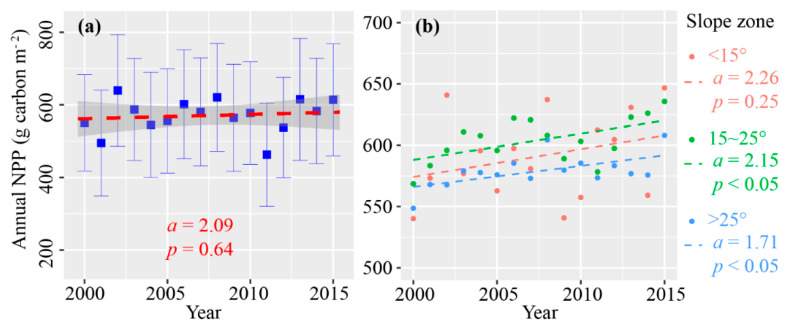
Temporal variation of annual NPP for the whole TGR area (**a**) and for different slope zones (**b**). Note: The dotted line indicates the linear fitting from 2000 to 2015, the a describes the changing trend. Meanwhile, the t statistic was applied to test the significance of the modeled *slope*, and the *p* value documents the significance. In the figure (**a**), error bars extending from the means document the standard deviation of Annual NPP, while the gray region is the 95% confidence intervals of the linear model.

Annual NPP of the TGR area increased from 547.40 gC•m^−2^ in 2000 to 629.96 gC•m^−2^ in 2015. During this period, the maximum occurred in 2002 with the annual NPP of 645.73 gC•m^−2^, while the minimum occurred in 2011 with the annual NPP of 462.84 gC•m^−2^. According to the trend analysis of the NPP time series, annual NPP of the TGR area presented an increasing trend with the rate of 2.09 gC•m^−2^•yr^−1^. However, the changing trend of annual NPP of the TGR area was not significant because the *p*-value of *t*-test for the modeled *slope* was far more than 0.05 (Figure 2a).

Meanwhile, the change trends of annual NPP varied among three slope zones (Figure 2b). The slopes of <15°, 15~25°, and >25° accounted for 46.23%, 29.03%, and 24.74% of the total area in the TGR area, respectively. Although the annual NPP of the zone of slope <15° increased at a rate of 2.26 C•m^−2^•yr^−1^, it was not statistically significant. The annual NPP increased significantly in the zones of 15~25° and >25°, with the rates of 2.15 C•m^−2^•yr^−1^ and 1.71 C•m^−2^•yr^−1^, respectively.

### 3.2. Spatial Variation of Annual NPP in the TGR Area

**Figure 3:**
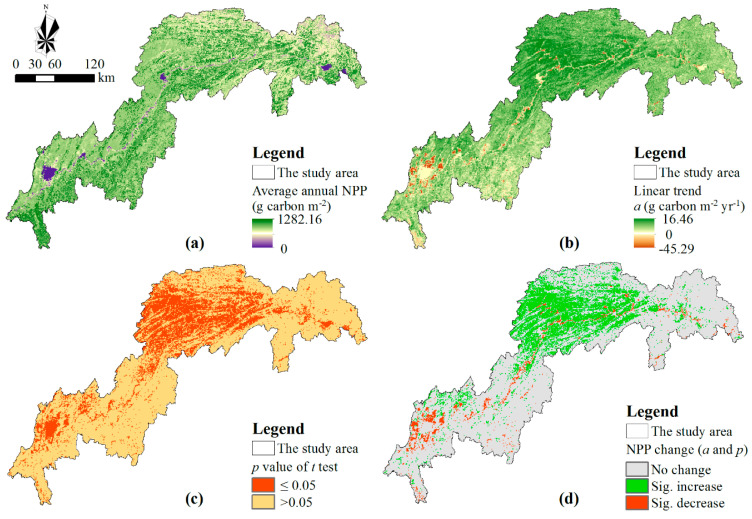
Spatiotemporal variations of annual NPP in the TGR area at the pixel level. Average annual NPP (**a**) change trends of annual NPP; (**b**), the *p*-value of t-test for the modeled *slopes*; (**c**), and the NPP change types (**d**). Note: The least-square linear regression model was applied to analyze the temporal variation of annual NPP from 2000 to 2015 for each pixel, and the changing trend is described by the modeled *slope* which is recorded by a in the figure (**b**). In the figure (**d**), No change documents that annual NPP did significantly no change (a = 0 and *p* ≤ 0.05) or changed but not significantly (a ≠ 0 and *p* > 0.05). Sig. increase documents annual NPP increased significantly (a > 0 and *p* ≤ 0.05), while Sig. decrease documents annual NPP decreased significantly (a < 0 and *p* ≤ 0.05).

To illuminate the spatial variation of annual NPP in the TGR area, the average and linear annual NPP trends were evaluated pixel by pixel from 2000 to 2015 (Figure 3). According to the average of annual NPP, the carbon sequestration exhibited a distinct spatial heterogeneity in the TGR area (Figure 3a). The average of annual NPP was lower in the southwest region than in the northeast region. Meanwhile, annual NPP increased slightly in the middle of the reservoir area, and the maximum increased rate was 16.46 gC•m^−2^•yr^−1^ (Figure 3b). Annual NPP showed a decreasing trend from 2000 to 2015 for the pixels around the built-up area and on both sides of the Yangtze River. In these regions, the maximum decreased rate was –45.29 gC•m^−2^•yr^−1^. Moreover, annual NPP changed significantly for the middle of the TGR area because the *p* values of *t*-test for the modeled *slope* of these pixels were less than 0.05 (Figure 3c).

According to the linear trends of annual NPP and their significances, three NPP change types were defined and illuminated (Figure 3d). Although 48.82% of the total area had an increasing trend for annual NPP, only 21.19% of the total area presented a significant increase in annual NPP (*p* < 0.5), and these pixels were distributed in the middle of the TGR area. Although 15.16% of the total area had the decreasing trend for annual NPP, only 3.60% of the TGR area presented a significant decrease in annual NPP (*p* < 0.5), mainly built-up areas and water in 2015. Consequently, 36.03% of the reservoir area showed no change in annual NPP at 0.05 statistically significant level, and 39.18% of the TGR area changed insignificantly for annual NPP during 2000–2015.

### 3.3. Effects of Climate and Land Use Changes on NPP Dynamics

**Figure 4:**
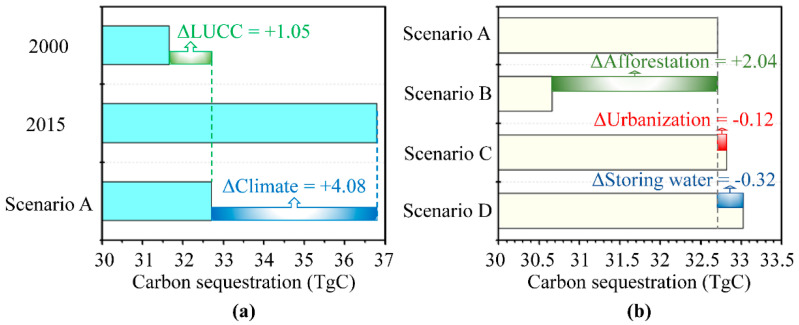
The effects of climate and all land use changes (**a**) and the effects of afforestation; urbanization and storing water (**b**) on NPP derived from annual carbon sequestration of the TGR area in 2000, 2015, and four scenarios. Note: ΔLUCC, the effect of all land use changes; ΔClimate, the effect of climate change; ΔAfforestation, the effect of afforestation; ΔUrbanization, the effect of urbanization; ΔStoring water, the effect of storing water. For each effect value (Δ), the number records the magnitude, while the plus or minus sign in front of the number indicates sequestrating or releasing carbon.

Based on the scenario A, we conducted the potential annual NPP of 2015 induced by all land use changes (NPP_A_) with the value of 565.52 gC•m^−2^. Converting annual NPP to annual carbon sequestration capacity, we knew the actual carbon sequestration capacities of the TGR area in 2000 and 2015 were 31.66 TgC (1Tg = 10^12^g) and 36.79 TgC, respectively (Figure 4a). The potential carbon sequestration capacity of the TGR area in 2015 was 32.71 TgC according to scenario A. Therefore, all land use changes could contribute to carbon sequestration capacity with 1.05 TgC, while climate changes could contribute to carbon sequestration capacity with 4.08 TgC.

Coupling the land use maps of 2000 and 2015, we reclassified these land use changes into the following four types (Figure S2): no change (79.8%), afforestation (15.5%), urbanization (2.3%), storing water (1.1%), and other land use changes (1.2%). Afforestation was scattered throughout the TGR area, while built-up land expanded dramatically in the southwest, especially in Chongqing City. Storing water submerged the low-lying areas along the Yangtze River. In light of scenario B, afforestation was positively related to carbon sequestration, and could sequestrate carbon with the capacity of 2.04 TgC for the whole TGR area (Figure 4b). However, urbanization and storing water both could release carbon with the capacity of 0.12 TgC and 0.32 TgC for the whole TGR area according to scenarios C and D. Meanwhile, we used the Equation (11) to calculate the effect of other land use changes and converted it into a carbon sequestration capacity of −0.55 TgC, indicating other land use changes could also release carbon.

### 3.4. Spatial Variations of the Individual Effect of Land Use Change

**Figure 5:**
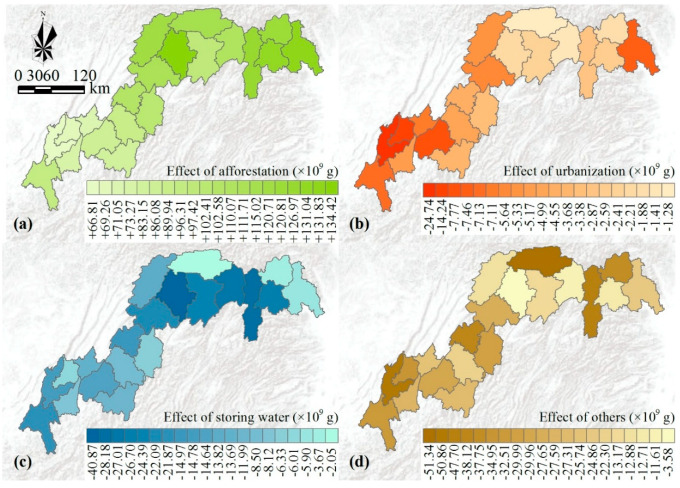
Spatial variations of the effects of afforestation (**a**); urbanization (**b**); storing water (**c**), and other land use changes (**d**) on NPP change at the county level.

According to four scenarios, we examined the effects of four land use changes on the NPP dynamics for 20 counties to illuminate the spatial variations (Figure 5). Overall, the combined effects of all land use changes would contribute to carbon sequestration in most counties except Chongqing. Afforestation made a great contribution to carbon sequestration and storage. The effect of afforestation on NPP dynamics was greater in the east-north reaches than in the west-south reaches (Figure 5a). Carbon sequestration capacities caused by afforestation were more than 100 GgC (1Gg = 10^9^g) for these counties in east-north. The maximum was 134.42 GgC in the Yunyang, while the minimum was 66.81 in Chongqing.

Urbanization would release carbon, and the individual effect exhibited a distinct spatial heterogeneity (Figure 5b). Chongqing presented the greatest effect with −24.74 GgC, much higher than Yubei, which showed the second greatest effect with −14.24 GgC. The effect was low in the middle reach of the TGR area and was only −1.28 GgC in Wuxi. Storing water would also release carbon, and the individual effect was larger for these counties along the Yangtze River (Figure 5c). Yunyang presented the greatest effect with the value of −40.87 GgC, while Wuxi presented the smallest effect with the value of −2.05 GgC. Meanwhile, other land use changes would release many carbons, and the capacity fluctuated between −3.58 GgC and −51.34 GgC (Figure 5d).

## 4. Discussion

### 4.1. Change in NPP of the TGR Area from 2000 to 2015

According to the CASA model’s estimation, annual NPP of the TGR area presented an increasing trend (Figure 2a). The average annual NPP was about 550 gC•m^−2^ which was in agreed with other relevant studies [30,43]. Despite a lack of experimental validation, we used the correlation analysis to validate the estimation accuracy because the vegetation productivity was usually closely related to aboveground biomass [32]. Our estimated results are supported by the significantly strong positive relationship between the aboveground biomass and the NPP estimation (Figure S1).

The estimated NPP of the TGR area in our study agreed with relevant studies (such as Zeng et al. [43]) based on field experiments and a statistical model. Moreover, the spatiotemporal dynamics of annual NPP were consistent with other process-based model simulations. For instance, Chen and Xiao [30] used the Biome-BGC model to assess the NPP for different forests of the TGR area, and they reported that annual NPP showed an increasing trend during 1992–2012 and the average NPP value was 550.29 gC•m^−2^.

### 4.2. Possible Effects of Climate Changes

**Figure 6:**
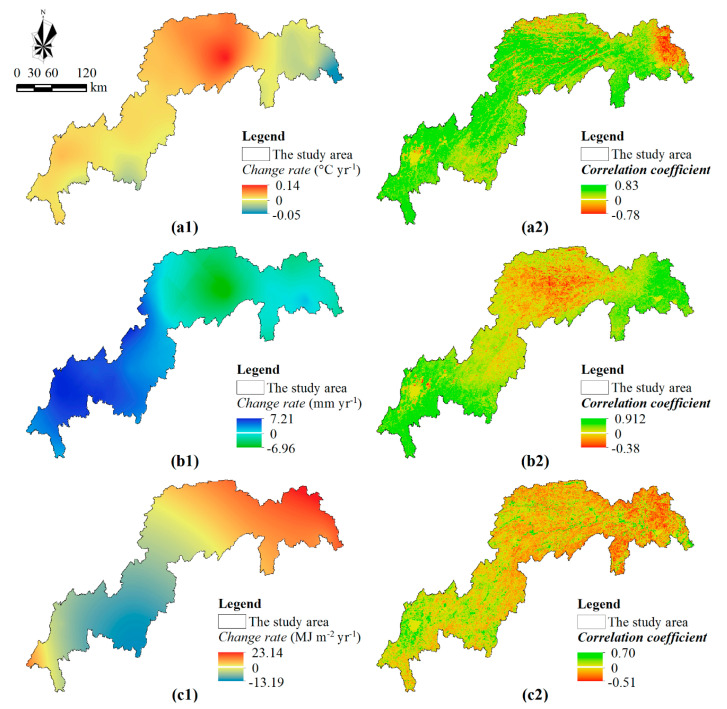
Spatial variation of climate variables and Spearman’s rank correlations between the annual NPP and these variables for the period 2000–2015. Note: The least-square linear regression model was applied to analyze the temporal variations of these climate variables for each pixel, and the change trend is described by the modeled *slope* in the figures of (**a1**,**b1**,**c1**). The Spearman’s rank correlation coefficient was applied to analyze the relationship between NPP and climate variable in the figures (**a2**,**b2**,**c2**).

NPP is a sensitive indicator of climate change [12], and climate change could help to sequestrate 4.08 Tg carbon for the whole TGR area between 2000 and 2015 (Figure 4a). The annual mean temperature of the TGR area was higher in 2015 (17.37 °C) than in 2000 (16.47 °C), and presented an insignificant increasing trend with the change rate of 0.03 °C/yr according to the change trend analysis (Figure S3(a1)). Meanwhile, the annual mean temperature had an increasing trend in most regions, especially in the middle reach of the TGR area (Figure 6(a1)). Temperature is a key factor affecting NPP, and some studies (such as Reyer et al. [29], Langerwisch et al. [19], Zhang et al. [45]) suggested NPP would increase with temperature because a warmer climate helps to enhance photosynthesis for vegetation growth, but some studies have indicated that NPP would decrease with temperature because a warmer climate helps to increase plant’s autotrophic respiration [46,47]. Our results supported the former insight. Spearman’s rank correlation between NPP and annual mean temperature was significant, and the correlation coefficient was 0.55 (Figure S3(a2)). The spatial explicit correlation coefficients were positive in most regions, and the maximum was 0.83 (Figure 6(a2)). Therefore, climate warming could partially explain the increasing NPP, and the increasing temperature may have a significant positive effect on the annual NPP of the TGR area in the future.

As an important climatic factor, precipitation plays a key role in vegetation productivity [47]. According to the temporal variation (Figure S3(b1)), the TGR area’s annual rainfalls were 1211.47 mm and 1181.34 mm in 2000 and 2015, respectively. The changing trend of annual rainfall exhibited significant heterogeneity (Figure 6(b1)). In the TGR area, the annual rainfall increased significantly in the west-south reach but decreased significantly in the east-north reach. Annual NPP was positively correlated with annual rainfall, and the Spearman’s rank correlation coefficient was 0.49 (Figure S3(b2)). Moreover, the relationship between annual NPP and rainfall was positively in most regions but was negatively in the middle reach of the TGR area (Figure S3(b2)). It was not surprising to the positive relationship between annual NPP and rainfall because precipitation could improve plant photosynthesis [48]. However, the negative effect of rainfall on the NPP dynamics in the middle reach was difficult to explain. The topography of this reach was complex and rugged, and precipitation was higher in the TGR area, which could increase soil erosion according to our previous studies [36,49]. Soil loss could reduce soil organic matter content and indirectly reduce plant’s productivity, which also was supported by other studies [9,50,51], especially in the high-precipitation regions. Therefore, soil conservation caused by declining rainfall may be partially responsible for the increasing NPP in the middle reach.

The TGR area’s annual radiation increased from 4151.651 MJ•m^−2^ in 2000 to 4380.987 MJ•m^−2^ in 2015, and the change rate was 15.28 MJ•m^−2^/yr (Figure S3(c1)). Meanwhile, annual radiation showed an increasing trend in the east-north reach, but a decreasing trend in the west-south reach (Figure 6(c1)). Radiation was the only energy source, and the NPP dynamic usually was positively related to radiation. However, the correlation between annual NPP and radiation was weaker in our study, and the Spearman’s rank correlation coefficient was only 0.17 (Figure S3(c2)). Meanwhile, NPP increased with radiation in most regions but decreased with radiation in some regions (Figure 6(c2)). According to the Spearman’s rank correlation, most relationships between annual NPP and radiation were not significant. Therefore, we guessed the relative effect of radiation was weaker than that of temperature and rainfall on the NPP dynamic because the TGR area has a humid mid-subtropical monsoon climate with rich light and the radiation was not the limiting factor for plant’s production [9,31]. Moreover, not only changes in climatic conditions but also atmospheric carbon dioxide (CO_2_) concentrations, aerosol concentration, atmospheric factors, and other factors have been highlighted as possible causes of NPP dynamic [6,21,25], the composite effect of these factors could alter the absorbed photosynthetic active radiation and then affect the relationship between annual NPP and radiation.

### 4.3. Possible Effects of Land Use Changes

**Figure 7:**
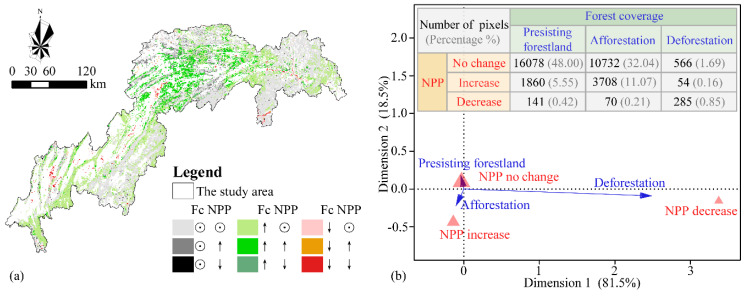
Spatial coupling map (**a**) and correspondence analysis; (**b**) between NPP change and forest change. Note: In the figure (**a**), Fc is an acronym for forest coverage, while ⊙, ↑ and ↓ indicate no change, increase, and decrease, respectively.

To better document the responses of NPP to different land use changes in the TGR area, we used four simulation scenarios to decouple the combined and individual effects of land use changes (Figure 4b). The combined effect of all land use changes could sequestrate carbon with an annual capacity of 1.05 TgC, and afforestation made a considerable contribution. Forests played the most critical role in terrestrial carbon accumulation [6,8,51]. This could explain the positive contribution of the implementation of green infrastructure projects on annual NPP of the TGR area. Moreover, we used the 1km grid to map the forest coverage change and annual NPP change and discussed their spatiotemporal relationships (Figure S4). The spatial coupling map between forest change and NPP change (Figure 7a) revealed that the regions with an increasing tendency of NPP and forest coverage were mainly concentrated in the middle of the TGR area, while that with a decreasing tendency mostly were cities and water areas. The grid number of both persisting forestland and no change in NPP was larger, and their relationship was strongly positive according to the correspondence analysis (Figure 7b). Therefore, we could conclude that afforestation and deforestation could partially explain the increase and decrease of annual NPP in the TGR area because of the spatiotemporal consistencies between NPP change and forest coverage change.

In a previous study [1], we have reported that deforestation was mainly caused by urbanization and storing water in the TGR area. We assessed their individual effects, and they could release 0.12 Tg and 0.32 Tg carbon for the whole TGR area, respectively (Figure 4b). According to the spatial variations, the individual effect of urbanization and storing water presented significantly heterogeneity (Figure 5). In detail, releasing carbon caused by urbanization was higher in the west-south than in the east-north, coinciding with these human activities about urban construction such as Western Development Project (Figure 5b). Meanwhile, releasing carbon caused by storing water was higher in the east-north than in the west-south. This is large because these east-north counties were more severely affected by the Three Gorges Dam (Figure 5c). Therefore, storing water and urbanization occurred in different regions, which led to the distinct spatial heterogeneity of potential carbon losses.

Other land use changes such as degradation from forests to grasses only accounted for 1.2% of the TGR area and would release 0.55 Tg carbon (Figure 5d). A series of ecological problems have seriously influenced terrestrial ecosystems due to the complex human activities and the rugged topography [38,39], leading to land degradation and desertification in the reservoir area. Although other land use changes would bring adverse ecological effects and release carbon, the Chinese government has employed many ecological restoration policies since 2000, which could offset these negative effects [20,35]. Thus, green infrastructure projects should be maintained and implemented adequately and sustainably in the future and play a greater role in offsetting potential carbon losses.

### 4.4. Land Use Policies and Implications

Over the decades, many green infrastructure projects have been implemented in China to improve the ecosystem structure [1,20,35]. However, human activities such as urbanization expansion and the hydropower construction of the Three Gorges Dam have accelerated the degradation of ecosystem functions [1]. Multiple land use changes presented confounded, and interactive effects on NPP dynamics [9,19]. The combined effect of all land use changes would effectively improve carbon sequestration in the TGR area (Figure 5a). Our results demonstrated that afforestation could positively promote terrestrial carbon sequestration by enhancing vegetation coverage, and offset carbon losses caused by human activities such as urbanization and storing water. On one hand, afforestation converted the degraded land or sloping cropland to forestation, increasing vegetation production in the unit and the efficiency of terrestrial carbon sequestration [20,51]. On the other hand, the magnitude of the positive effect caused by vegetation restoration was larger than the negative effect caused by urbanization and other economic activities because the green infrastructure projects always were implemented on a larger scale with a long duration. Therefore, although multiple land use changes could occur simultaneously and would bring both positive and negative ecological effects, the implementation of green infrastructure projects such as afforestation could partly offset the negative ecological effect.

**Figure 8:**
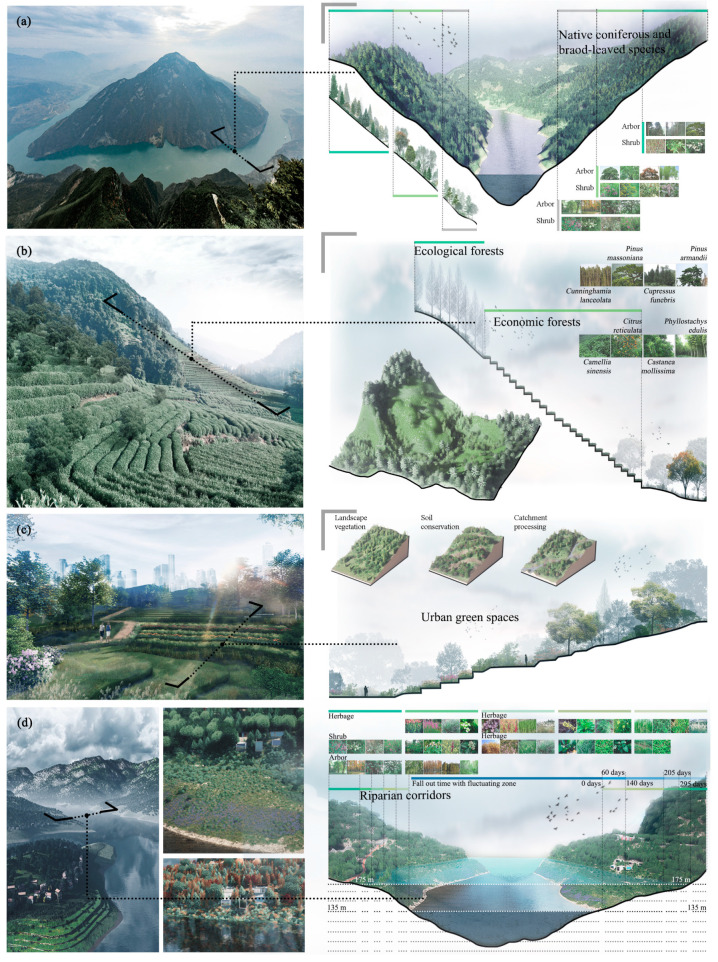
Green infrastructure planning and designing for ecological forest landscape (**a**); agroforestry landscape (**b**); urban green space (**c**) and riparian corridor (**d**) in the TGR area. Note: The left pictures illuminate the complex topographical characters and various landscapes, while the right pictures are our planning and designing sections of green infrastructure for these landscapes.

To achieve regional sustainable development, it is a vital issue for planning and designing the efficient green infrastructure [52,53]. Forest landscapes in mountains usually have strong ecological functions, because these green infrastructures mainly consist of native coniferous and broad-leaved species with faster growth and strong adaptability and would provide larger carbon sequestration capacity. The section of the forest landscape presented that the broad-leaved forests are in the low altitude, the mixed forests are in the middle altitude, and the coniferous forests are in the high altitude (Figure 8a). Meanwhile, agroforestry landscapes could provide both economic values and ecosystem services which differ in quantity and quality from conventional agricultural practices, and consist of ecological forests and economic forests such as tea gardens and orchards in the TGR area (Figure 8b). Urban green spaces have ecological, social, and economic functions, and could provide multiple or cross-cutting services such as recreation, aesthetics and air purification (Figure 8c). The special riparian corridors usually consist of shrubs and herbs in the TGR area, and this landscape is an ecotone between aquatic and terrestrial ecosystems (Figure 8d). Because the TGR area has the complex topographical characters and various landscapes, it is urgent and important to provide more proper and feasible landscape planning schemes of green infrastructure for the corresponding landscape in the future.

### 4.5. Study Limitations

Remote sensing data and the NPP model could help us understand the spatiotemporal variations of terrestrial NPP at the regional scale. Nevertheless, due to the inaccuracies of data and the inherent limitations of the CASA model [31,33], uncertainties still exist. First, the spatial maps of temperature, precipitation, and radiation were generated by geospatial interpolation from the climatic observations of meteorological stations, and uncertainties are inevitable. The second source of uncertainty for modeled NPP came from the downscaling of MODIS-NDIV products. Third, an increasing number of studies demonstrated that atmospheric processes, such as increasing atmospheric carbon dioxide concentrations [24,25] and nitrogen deposition [23,54], would affect the plant’s production. As a common issue, the CASA model takes account of some climatic factors but not atmospheric factors, which is a limitation for NPP estimation.

Although there were some limitations and uncertainties for assessing NPP, all of the studies reviewed so far note it as the greatest challenge. Moreover, our study focused more on the spatiotemporal variations than the accuracy of estimated NPP, and we believed the model’s estimation accuracy was to meet our demand.

## 5. Conclusions

Although critical for estimating various land use changes and their ecological effects, previous studies have failed to decouple the respective and the combined effects of different land use changes on ecosystem services. Based on the estimation of CASA model, the terrestrial NPP of the TGR area presented an increasing trend during 2000–2015. Meanwhile, we conducted that climate change and land use change both could contribute to carbon sequestration in the TGR area according to the scenario design. However, land use changes could bring both positive and negative ecological effects simultaneously over the past decades. Among these land use changes, only afforestation could sequestrate carbon, indicating that green infrastructure had effectively offset the carbon emissions. Although urbanization and storing water both released carbon, these land use changes and their negative ecological effects exhibited significantly spatial heterogeneity. Urbanization-induced carbon emission was larger in the southwest than in the northeast, while impoundment-induced carbon emission was larger for these counties along the Yangtze River. Green infrastructure has effectively offset the carbon emissions from urbanization and storing water in the TGR area, which provides some fundamental supports for further ecological restoration and contributes to empowering land use policies towards carbon sequestration and storage at the regional scale.

## Figures and Tables

**Table 1:** The study data sources and their explanations.

Data	Data Source and Explanation
Land use data	30m resolution land use maps in 2000, 2005, 2010, and 2015 [35].
Radiation data	Monthly total radiation data of 22 meteorological stations from 2000 to 2015 (http://data.cma.cn)
Precipitation and temperature data	Monthly total precipitation and mean temperature data of 29 meteorological stations from 2000 to 2015 (http://data.cma.cn)
NDVI images	250m resolution MODIS-NDVI products (MOD13Q1) from 2000 to 2015 (http://modis.gsfc.nasa.gov/)
DEM	30m resolution DEM derived from ASTER Global Digital Elevation Model V002 (http://www.gscloud.cn/)

**Table 2:** Parameters of the CASA model for different land use types.

Land Use Type	NDVI*_max_*	NDVI*_min_*	SR*_max_*	SR*_min_*	ε*_max_*
Coniferous forest	0.8889	0.0765	17.002	1.166	0.389
Mixed forest	0.8852	0.0765	16.422	1.166	0.475
Evergreen broad-leaved forest	0.8979	0.0765	18.589	1.166	0.985
Deciduous broad-leaved forest	0.9058	0.0765	20.231	1.166	0.692
Shrubby	0.8983	0.0765	18.666	1.166	0.429
Grassland	0.7994	0.0765	8.970	1.166	0.542
Cropland	0.7994	0.0765	8.970	1.166	0.542
Water, built-up land, and bare land	0.7994	0.0765	8.970	1.166	0.542

## Supplementary Materials

The following are available online at https://www.mdpi.com/1660-4601/17/21/8077/s1, Table S1. Scenario design for quantifying the effects of climate change and land use changes on the NPP dynamic. Figure S1. The relationship between the aboveground biomass and the NPP estimation of CASA. Figure S2. Spatial explicit land use changes derived from land use maps of the TGR area in 2000 and 2015. Figure S3. Temporal variations of climate variables and Spearman’s rank correlations between annual NPP and these variables in the total area of the TGR area, China. Figure S4. Spatiotemporal variations of the forest coverage in the TGR area, China.
