# Supplementary material for: Green Infrastructure Offset the Negative Ecological Effects of Urbanization and Storing Water in the Three Gorges Reservoir Area, China
Source: Int J Environ Res Public Health. 2020 Nov 2;17(21):8077. doi: 10.3390/ijerph17218077 (PMC7663030; doi:10.3390/ijerph17218077)
Supplement: Supplementary file 1 [file ijerph-17-08077-s001.pdf]

**Table S1.** Scenario design for quantifying the effects of climate change and land use changes on the NPP dynamic.

| Scenario | Explanation/purpose |
|---|---|
| A | Keeping the climate conditions at the level of 2000, the potential NPP of 2015 ($NPP_A$) could be estimated by using the land use map and NDVI images of 2015. We could then calculate the effect of all land use/cover changes according to the equation: $\Delta LUCC = NPP_A - NPP_{2000}$. On the one hand, we knew that the overall effect equals the difference between the actual NPP of 2000 and 2015, i.e., $\Delta = NPP_{2015} - NPP_{2000}$. On the other hand, we hypothesized that the overall effect only consisted of the effects of climate change and land use changes, i.e., $\Delta = \Delta Climate + \Delta LUCC$. Therefore, we could also calculate the effect of climate change using the equation: $\Delta Climate = NPP_{2015} - NPP_A$. |
| B | Keeping the climate conditions and the NDVI values for afforestation pixels as the level of 2000, we could calculate the potential NPP of 2015 caused by land use changes except for afforestation ($NPP_B$). Then, we could calculate the effect of afforestation according to the equation: $\Delta Afforestation = NPP_A - NPP_B$. |
| C | Keeping the climate conditions and the NDVI values for urbanization pixels as the level of 2000, we could calculate the potential NPP of 2015 caused by land use changes except for urbanization ($NPP_C$). Then, we could calculate the effect of urbanization according to the equation: $\Delta Urbanization = NPP_A - NPP_C$. |
| D | Keeping the climate conditions and the NDVI values for storing water pixels as the level of 2000, we could calculate the potential NPP of 2015 caused by land use changes except for storing water ($NPP_D$). We then could calculate the effect of storing water according to the equation: $\Delta Storing\ water = NPP_A - NPP_D$. |

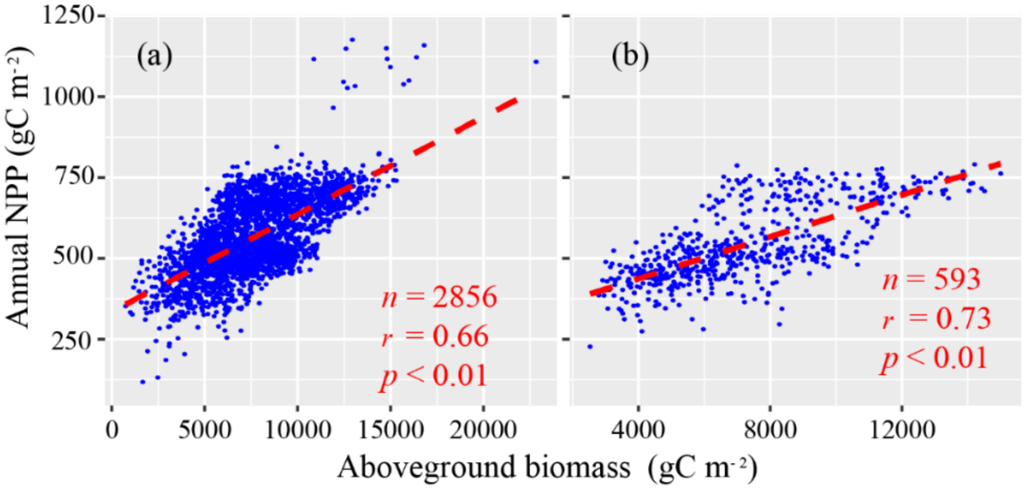


**Figure S1.** The relationship between the aboveground biomass and the NPP estimation of CASA in 2000 **(a)** and 2013 **(b). Note:** *n* is the number of forest plots, *r* is the Pearson correlation coefficient, and *p* is used to identify the significance of correlation.

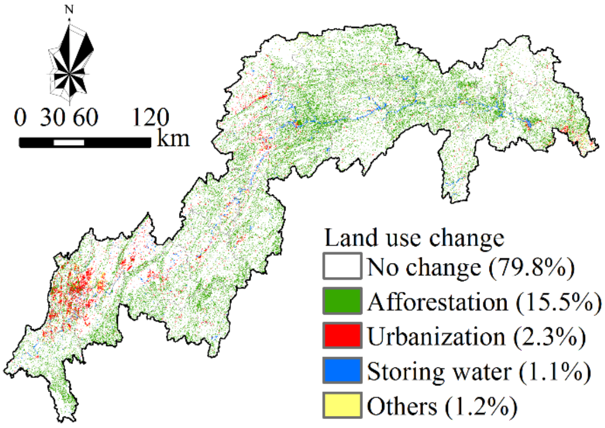


**Figure S2.** Spatial explicit land use changes derived from land use maps of the TGR area in 2000 and 2015.

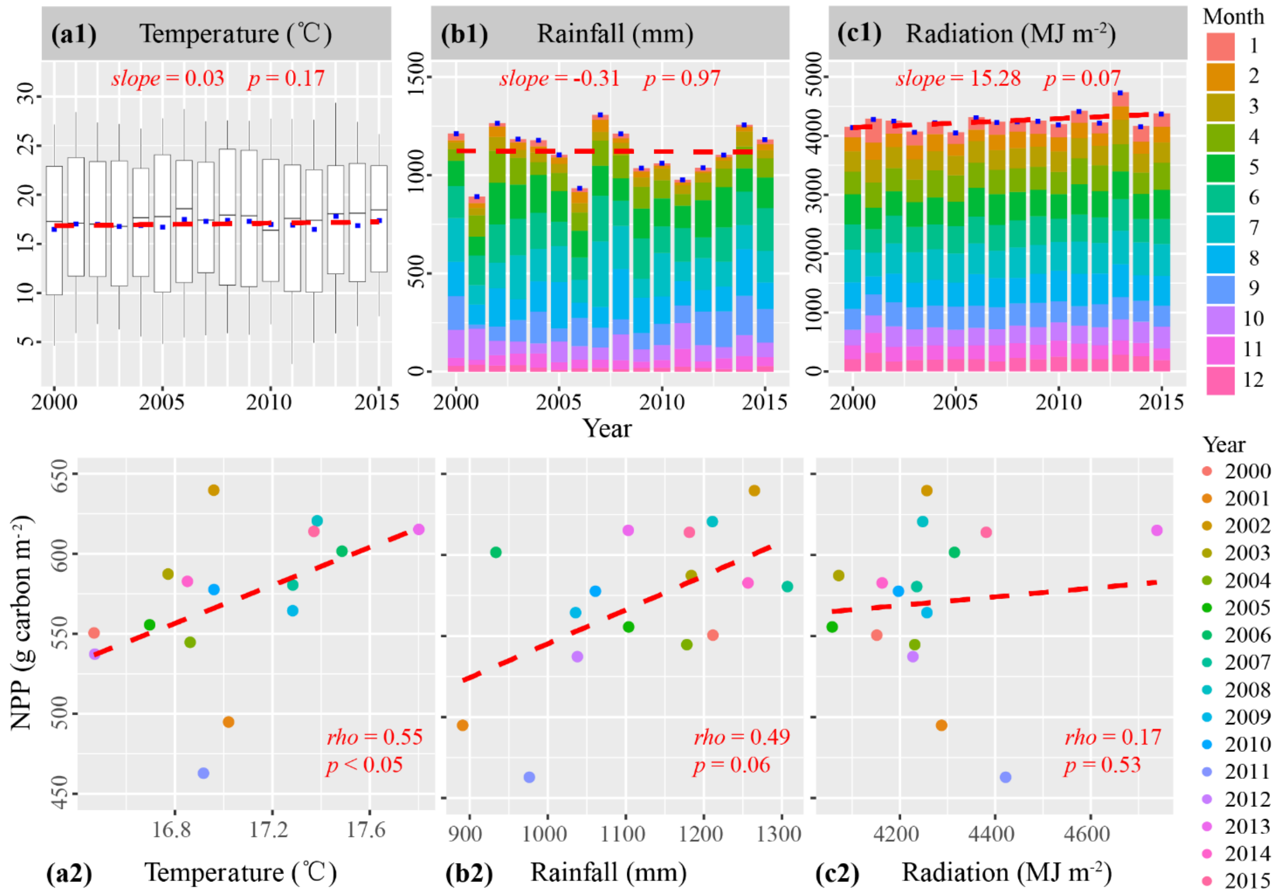


**Figure S3.** Temporal variations of climate variables and Spearman's rank correlations between annual NPP and these variables in the total area of the TGR area, China. **Note:** In the figures of (a1), (b1) and (c1), the red dotted line indicates the linear fitting during the period of 2000 to 2015, the *slope* describes the changing trend of the climate variable, and *p* value of *t* statistic is used to identify the significance of the changing trend. In the figures of (a2), (b2) and (c2), red dotted line indicates the linear relationship between NPP and climate variable, *rho* is the correlation coefficient, and *p* value is used to identify the significance of the correlations.

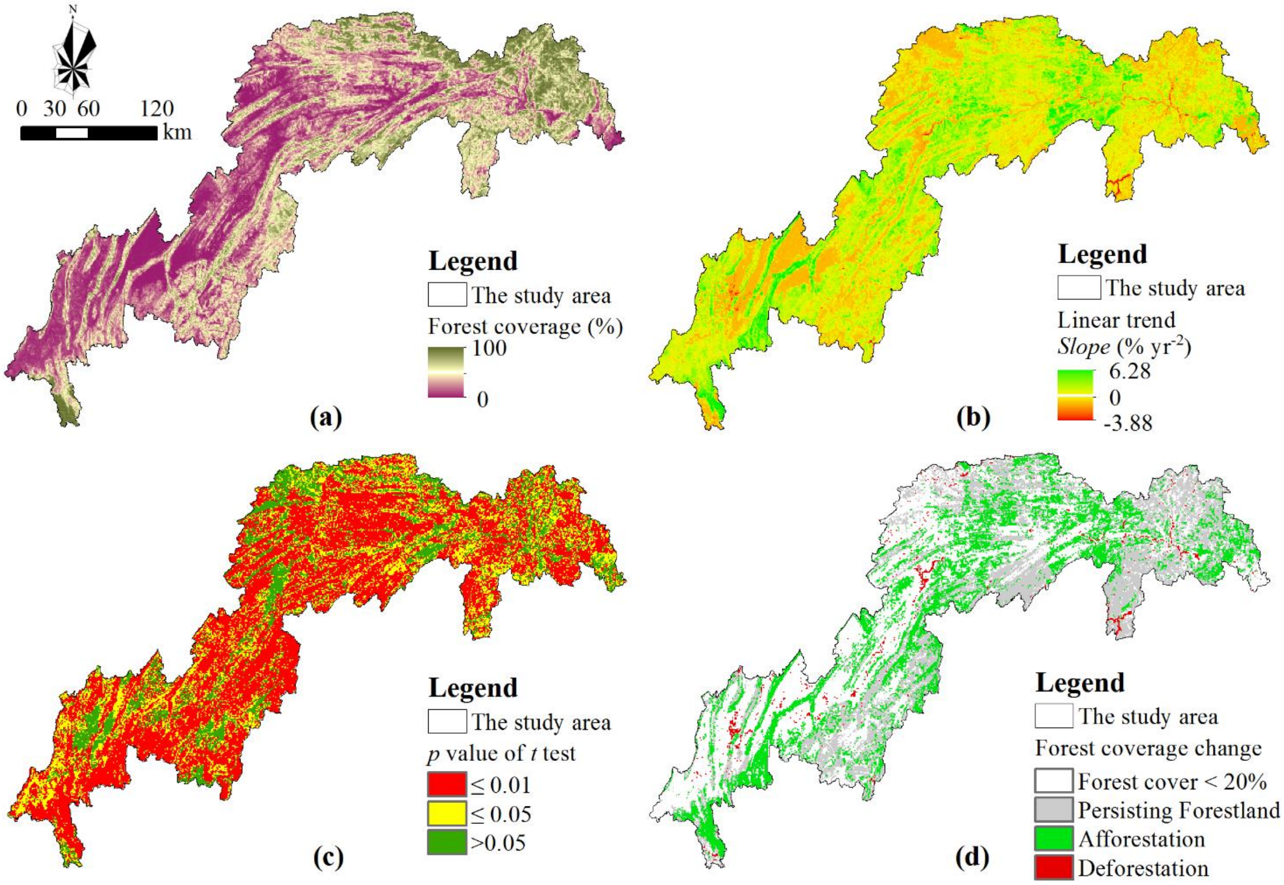


**Figure S4.** Temporal and spatial variation of the forest coverage in the TGR area, China. The average forest coverage (a), *slope* of linear model for the forest coverage change (b), $p$ value of $t$ test for the *slope* (c) and forest coverage change types (d) in grids (1km × 1km). **Note:** in the figure (d), the grid with average forest coverage less than 20% was set the null. Persisting forestland documented that the forest coverage did significantly no change (slope = 0 and $p \leq 0.05$) or changed but not significantly (slope ≠ 0 and $p > 0.05$) in the grid with average forest coverage more than 20%. Afforestation documented that the forest coverage increased significantly (slope > 0 and $p \leq 0.05$), while deforestation documented that the forest coverage decreased significantly (slope < 0 and $p \leq 0.05$).
